# SEX/GENDER DIFFERENCES IN DIAGNOSIS OF PAIN AMONG OLDER MEDICARE BENEFICIARIES

**DOI:** 10.1093/geroni/igae098.1810

**Published:** 2024-12-31

**Authors:** Sadaf Milani, Yong Shan, Yong-Fang Kuo, Mukaila Raji

**Affiliations:** The University of Texas Medical Branch, Galveston, Texas, United States; The University of Texas Medical Branch, Galveston, Texas, United States; University of Texas Medical Branch, Galveston, Texas, United States; University of Texas Medical Branch, Galveston, Galveston, Texas, United States

## Abstract

Pain is often underdiagnosed among women, especially Black women, but the extent of underdiagnosis is unknown. Cognitive status can also complicate pain reporting and diagnosis. Our objective was to assess the odds of having a pain diagnosis, by sex/gender, while considering race/ethnicity and cognitive status. We used data from the 2018 Health and Retirement Study linked to Medicare claims. We included individuals with complete 2018 Part A and B enrollment and complete information on covariates, excluding those with HMO. Our outcome combined self-reported frequent pain and diagnosis of chronic pain: no pain, self-report only, and diagnosed (both diagnosed only and diagnosed + self-report). Our exposure was sex/gender. We used multinomial logistic regression models to assess the odds of pain diagnosis, by sex/gender, controlling for age, education, race/ethnicity, rurality, cognitive status, and Elixhauser comorbidity index. Among our participants (n=3,523), 12.0% had no pain, 4.6% had undiagnosed pain, and 83.4% had diagnosed pain. Compared to men, women had higher odds of diagnosed pain. The interaction between gender and race/ethnicity was significant (p=0.02). When stratified by sex/gender, non-Hispanic (NH) Black women had lower odds of diagnosed pain, compared to NH White women. Compared to NH Black and Hispanic men, NH White men had lower odds of any type of pain (both diagnosed and self-reported). The main effect of cognitive status and interaction with gender was not significant. Contrary to our hypothesis, women had higher odds of diagnosed pain than men. Within racial/ethnic group differences exist in men and women and warrant future studies.

